# Hospital Characteristics and Early Enrollment Trends in the American College of Cardiology Voluntary Public Reporting Program

**DOI:** 10.1001/jamanetworkopen.2021.47903

**Published:** 2022-02-10

**Authors:** Yulanka S. Castro-Dominguez, Jeptha P. Curtis, Frederick A. Masoudi, Yongfei Wang, John C. Messenger, Nihar R. Desai, Lara E. Slattery, Gregory J. Dehmer, Karl E. Minges

**Affiliations:** 1 Harrington Heart and Vascular Institute, University Hospitals and Case Western Reserve University School of Medicine, Cleveland, Ohio; 2 Section of Cardiovascular Medicine, Department of Internal Medicine, Yale School of Medicine, New Haven, Connecticut; 3 Center for Outcomes Research and Evaluation, Yale-New Haven Hospital, New Haven, Connecticut; 4 Division of Cardiology, Department of Medicine, University of Colorado School of Medicine, Aurora; 5 American College of Cardiology, Washington, District of Columbia; 6 Carilion Clinic and Virginia Tech Carilion School of Medicine, Roanoke, Virginia; 7 Department of Health Administration and Policy, University of New Haven, West Haven, Connecticut

## Abstract

**Importance:**

Limited data exist regarding the characteristics of hospitals that do and do not participate in voluntary public reporting programs.

**Objective:**

To describe hospital characteristics and trends associated with early participation in the American College of Cardiology (ACC) voluntary reporting program for cardiac catheterization-percutaneous coronary intervention (CathPCI) and implantable cardioverter-defibrillator (ICD) registries.

**Design, Setting, and Participants:**

This cross-sectional study analyzed enrollment trends and characteristics of hospitals that did and did not participate in the ACC voluntary public reporting program. All hospitals reporting procedure data to the National Cardiovascular Data Registry (NCDR) CathPCI or ICD registries that were eligible for the public reporting program from July 2014 (ie, program launch date) to May 2017 were included. Stepwise logistic regression was used to identify hospital characteristics associated with voluntary participation. Enrollment trends were evaluated considering the date *US News & World Report* (*USNWR*) announced that it would credit participating hospitals. Data analysis was performed from March 2017 to January 2018.

**Main Outcomes and Measures:**

Hospital characteristics and participation in the public reporting program.

**Results:**

By May 2017, 561 of 1747 eligible hospitals (32.1%) had opted to participate in the program. Enrollment increased from 240 to 376 hospitals (56.7%) 1 month after the *USNWR* announcement that program participation would be considered as a component of national hospital rankings. Compared with hospitals that did not enroll, program participants had increased median (IQR) procedural volumes for PCI (481 [280-764] procedures vs 332 [186-569] procedures; *P* < .001) and ICD (114 [56-220] procedures vs 62 [25-124] procedures; *P* < .001). Compared with nonparticipating hospitals, an increased mean (SD) proportion of participating hospitals adhered to composite discharge medications after PCI (0.96 [0.03] vs 0.92 [0.07]; *P* < .001) and ICD (0.88 [0.10] vs 0.81 [0.12]; *P* < .001). Hospital factors associated with enrollment included participation in 5 or more NCDR registries (odds ratio [OR],1.98; 95% CI, 1.24-3.19; *P* = .005), membership in a larger hospital system (ie, 3-20 hospitals vs ≤2 hospitals in the system: OR, 2.29; 95% CI, 1.65-3.17; *P* = .001), participation in an NCDR pilot public reporting program of PCI 30-day readmissions (OR, 2.93; 95% CI, 2.19-3.91; *P* < .001), university affiliation (vs government affiliation: OR, 3.85, 95% CI, 1.03-14.29; *P* = .045; vs private affiliation: OR, 2.22; 95% CI, 1.35-3.57; *P* < .001), Midwest location (vs South: OR, 1.47; 95% CI, 1.06-2.08; *P* = .02), and increased comprehensive quality ranking (4 vs 1-2 performance stars in CathPCI: OR, 8.08; 95% CI, 5.07-12.87; *P* < .001; 4 vs 1 performance star in ICD: OR, 2.26; 95% CI, 1.48-3.44; *P* < .001) (C statistic = 0.829).

**Conclusions and Relevance:**

This study found that one-third of eligible hospitals participated in the ACC voluntary public reporting program and that enrollment increased after the announcement that program participation would be considered by *USNWR* for hospital rankings. Several hospital characteristics, experience with public reporting, and quality of care were associated with increased odds of participation.

## Key Points

**Question:**

What trends and hospital-related factors characterize enrollment in the voluntary public reporting program from the American College of Cardiology (ACC)?

**Findings:**

In this cross-sectional study reporting the early experience of the ACC voluntary public reporting program for catheterization and percutaneous coronary intervention (CathPCI) and implantable cardioverter-defibrillator (ICD) registries, one-third of 1747 eligible hospitals participated in the program. Enrollment increased by 57% after the announcement that program participation would be considered as a component of national hospital rankings.

**Meaning:**

These findings suggest that further work is needed to investigate benefits associated with fair and accurate public reporting and to identify hospital barriers to publicly reporting outcomes.

## Teaser

This cross-sectional study examines characteristics and early enrollment trends among hospitals participating in the American College of Cardiology (ACC) voluntary public reporting program.

## Introduction

Public reporting is intended to promote health care quality and transparency by providing consumers, payers, and clinicians and health care institutions access to information on hospital performance.^1,2^ Public reporting initiatives have proliferated, with the development of several national and state programs, as well as numerous efforts from payers, business consumer groups, and independent organizations.^3,4,5^ Many public reporting programs rely on administrative data, which limits clinical validity.^4,6,7^ Other programs report data generated with proprietary methods that are not disclosed.^5,8^ In comparison, clinical registry data are infrequently used for public reporting owing to the effort required to collect data; however, these data consider the nuances associated with delivering guideline-concordant care.^9^

Recognizing the barriers to clinically valid and meaningful reporting, the American College of Cardiology (ACC) initiated a voluntary public reporting program in 2014 from the NCDR (National Cardiovascular Data Registry) cardiac catheterization-percutaneous coronary intervention (CathPCI) and implantable cardioverter-defibrillator (ICD) registries. The advantages associated with the ACC’s public reporting program are that it uses robust and accurate clinical data, calculates performance using a transparent methodology, and is continuously subject to improvement and oversight.^9,10^ The program displays hospital-level process-of-care measures that are endorsed by the National Quality Forum pertaining to the prescription of medications at discharge for PCI and ICD.^9,11^ Hospitals have 30 days to review their information before deciding to display their performance ratings for that cycle of reporting.^9^ Consumers can access hospital-specific data on the ACC’s CardioSmart Find Your Heart a Home website^12^ permitting individuals to search by hospital name and location and see the institutions’ public reporting status by registry. In 2016, *US News & World Report* (*USNWR*) announced a transparency component in the calculation of its cardiology and heart surgery specialty rankings. Hospitals could receive up to 3 percentage points by participating in public reporting programs maintained by the ACC and Society of Thoracic Surgeons (STS).^13,14^

The aims of this study were to explore early enrollment trends in the ACC voluntary public reporting program and investigate hospital characteristics associated with participation in the first 3 years of the program and considering inclusion of participation in *USNWR* rankings. This investigation may inform understanding of current trends of enrollment in public reporting and help identify opportunities to amplify adoption in voluntary quality reporting programs.

## Methods

This cross-sectional study was deemed exempt from review and informed consent by the institutional review board at the Yale School of Medicine because it used existing data with no patient identifiers. This study follows the Strengthening the Reporting of Observational Studies in Epidemiology (STROBE) reporting guideline for cross-sectional studies.

### Data

The hospitals in this study were already enrolled in the NCDR CathPCI or ICD registries. Hospitals were first able to privately review their performance based on information provided by the registry and then decide whether to participate in public reporting. For analysis of enrollment trends, we included all hospitals participating in NCDR CathPCI or ICD registries that were eligible for the public reporting program from July 2014 (ie, the program launch date) to May 2017. To be eligible for the public reporting program, hospitals must meet a minimum for number of procedures (25 procedures annually for the CathPCI Registry and 11 procedures annually for the ICD Registry) and must have at least 9 months of data submitted to the registry.^9^ We then obtained the number of hospitals that participated in the voluntary public reporting program during this period and the dates participation began. For evaluation of hospital characteristics, we limited our analysis to hospitals eligible for public reporting from 2014 to 2016 and compared those that participated in the voluntary public reporting program for CathPCI or ICD registries with those that did not enroll in either registry. The study period predates expiration of the Coverage with Evidence Development policy for ICDs that mandated submission of all Medicare cases into an approved clinical trial or the ICD Registry, therefore capturing most ICD procedures.

### Variables

We reviewed hospital characteristics, including hospital location (urban, suburban, or rural), bed size, US region (Midwest, Northeast, South, or West), hospital ownership (public, private, or university), teaching hospital status, PCI volume, ICD volume, and whether the hospital was part of a hospital system, and if so, the number of hospitals in the system. We also assessed variables relevant to prior public reporting experience, including number of NCDR registries in which the hospital participated, whether the hospital was located in a state with a mandated PCI public reporting program (ie, Massachusetts, New York, Pennsylvania, or Washington), duration of the contract with NCDR, and participation in an earlier NCDR pilot project to report 30-day readmissions after PCI. Finally, we included hospital adherence to guideline-recommended medications at time of discharge after PCI and ICD, as well as hospital ACC performance star rating (Table 1).^12^ ACC star ratings are derived from the hospital’s absolute performance score for each reported metric. A higher performance score means better performance on the metric; performance scores are converted into star ratings based on cut points that were deemed clinically relevant (ie, 1 star = less than 75%; 2 stars = 76%-89%; 3 stars = 90%-94%; and 4 stars = 95% or more).^9^

**Table 1.** Publicly Reported Performance Measures^a^

Registry	Performance measure
ICD	No. of new ICD implants
	ACEI and ARB therapy at discharge for patients receiving ICD implant with left ventricular systolic dysfunction
	β-blocker at discharge for patients receiving ICD implant with a previous myocardial infarction
	β-blocker at discharge for patients receiving ICD implant with left ventricular systolic dysfunction
	Composite of discharge medications (ACEIs or ARBs and β-blockers) among eligible patients receiving ICD implant
CathPCI	No. of PCI or angioplasty procedures
	Proportion of patients with aspirin prescribed at discharge
	Proportion of patients who received a stent with a P2Y12 inhibitor prescribed at discharge
	Proportion of patients with a statin prescribed at discharge
	Proportion of patients receiving PCI with aspirin, statin, or P2Y12 inhibitor (if eligible) prescribed at discharge

Abbreviations: ACEI, angiotensin converting enzyme inhibitor; ARB, angiotensin receptor blocker; CathPCI, catheterization and percutaneous coronary intervention; ICD, implantable cardioverter-defibrillator; PCI, percutaneous coronary intervention; P2Y12, platelet ADP P2Y12 receptor.

^a^ As reported on the CardioSmart website.^12^

### Statistical Analysis

**Figure 1.**
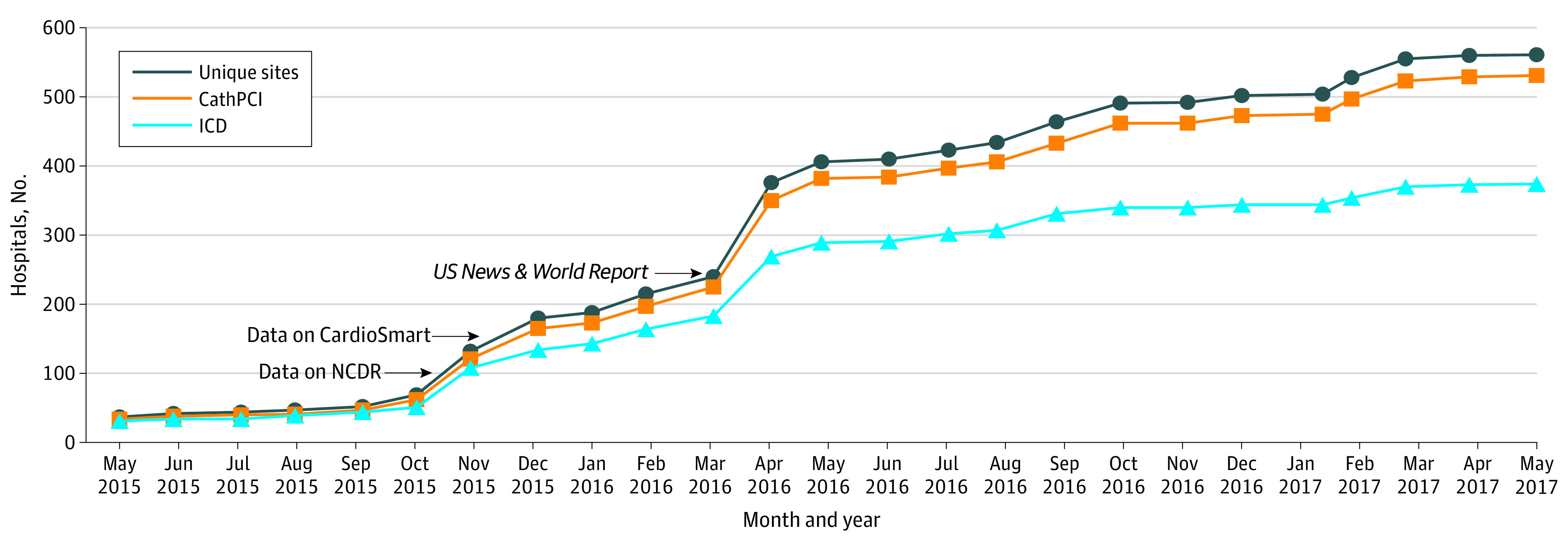
Voluntary Public Reporting Program Enrollment Trends, 2015 to 2017 NCDR indicates National Cardiovascular Data Registry^11^; data on NCDR, date when data were available privately for hospitals to review; data on CardioSmart,^12^ date when data were made public online; *US News & World Report,* date of announcement of credit for participation in public reporting by *US News & World Report*.

We calculated the proportion of eligible sites that enrolled in the voluntary public reporting program using monthly data from July 2014 to May 2017. We highlighted key dates relevant to public reporting, including the date data were available privately on the NCDR website for review, the date public reporting went live on the CardioSmart.org website, and the date of the *USNWR* announcement that it would credit hospitals participating in the ACC public reporting program. An enrollment trend graph was developed using these data (Figure 1).^11,12^

We compared characteristics of hospitals that enrolled in the public reporting program for at least 1 registry with those that did not enroll. Hospital adherence to guideline-recommended medications after PCI and ICD and performance star ratings were compared among participating and nonparticipating hospitals. Variables are presented as frequency with percentage, mean (SD), or median (IQR). Bivariate analysis was performed using χ^2^ test for categorical variables and *t* test for continuous variables, and tests were 2-sided. Variables with *P* < .05 were considered to be statistically significant and entered in the multivariable regression model. We used stepwise logistic regression to investigate hospital characteristics associated with participation in the voluntary public reporting program. Factors independently associated with participation in public reporting vs not participating in public reporting were included the multivariable model. Analyses were performed using SAS statistical software version 9.4 (SAS Institute). Data analysis was performed from March 2017 to January 2018.

## Results

### Hospital Characteristics

Characteristics of eligible hospitals that participated and hospitals that did not participate by the end of 2016 are displayed in Table 2. The 485 participating hospitals, compared with 1046 hospitals that did not enroll in public reporting, were more likely to be in an urban region (255 hospitals [52.6%] vs 447 hospitals [43.1%]; *P* < .001), university owned (76 hospitals [15.7%] vs 47 hospitals [4.5%]; *P* < .001), part of a hospital system (235 hospitals [48.5%] vs 427 hospitals [40.8%]; *P* < .001), and in the Midwest (158 hospitals [32.6%] vs 252 [24.2%]; *P* < .001) or Northeast regions (78 hospitals [16.1%] vs 111 hospitals [10.7%]; *P* < .001) and had an increased mean (SD) facility bed size (397 [247] beds vs 307 [186] beds; *P* < .001). Furthermore, participating hospitals had an increased median (IQR) volume of PCI procedures (481 [280-764] procedures vs 332 [186-569] procedures; *P* < .001) and ICD procedures (114 [56-220] procedures vs 62 [25-124] procedures; *P* < .001) and were also more likely to be enrolled in 5 or more NCDR registries compared with nonparticipating hospitals (142 hospitals [29.3%] vs 93 hospitals [8.9%]; *P* < .001). In addition, participating hospitals were more likely to be in states with mandatory public reporting programs (73 hospitals [15.1%] vs 100 hospitals [9.6%]; *P* = .002) and to have participated in the voluntary PCI 30-day readmission public reporting pilot program (204 hospitals [42.1%] vs 136 hospitals [13.0%]; *P* < .001).

**Table 2.** Hospital Characteristics

Characteristic	Hospitals, No. (%)			*P* value
	Total (N = 1531)^a^	Participating in public reporting		
		Yes (n = 485)	No (n = 1046)	
Location				
Rural	305 (20.0)	55 (11.3)	250 (24.1)	<.001
Suburban	516 (33.9)	175 (36.7)	341 (32.9)	
Urban	702 (46.1)	255 (52.6)	447 (43.1)	
Bed total, mean (SD)	336 (211)	397 (247)	307 (186)	<.001
Region				
Midwest	410 (26.9)	158 (32.6)	252 (24.2)	<.001
Northeast	189 (12.4)	78 (16.1)	111 (10.7)	
South	638 (41.8)	166 (34.2)	472 (45.4)	
West	288 (18.9)	83 (17.1)	205 (19.7)	
Hospital ownership				
Government	28 (1.8)	4 (0.8)	24 (2.3)	<.001
Private	1372 (90.1)	405 (83.5)	967 (93.2)	
University	123 (8.1)	76 (15.7)	47 (4.5)	
Teaching hospital	555 (36.4)	219 (45.2)	336 (32.3)	<.001
Part of a hospital system	662 (43.3)	235 (48.5)	427 (40.8)	.006
No. of hospitals in system				
≤2	869 (56.8)	250 (51.6)	619 (59.2)	<.001
3-20	315 (20.6)	144 (29.7)	171 (16.4)	
>20	347 (22.7)	91 (18.8)	256 (24.5)	
Volume of procedures, median (IQR)				
PCI	371 (207-642)	481 (280-764)	332 (186-569)	<.001
ICD	77 (29-155)	114 (56-220)	62 (25-124)	<.001
No. of registries enrolled				
2	438 (28.6)	77 (15.9)	361 (34.5)	<.001
3	587 (38.3)	170 (35.1)	417 (39.9)	
4	271 (17.7)	96 (19.8)	175 (16.7)	
≥5	235 (15.4)	142 (29.3)	93 (8.9)	
In public reporting state	173 (11.3)	73 (15.1)	100 (9.6)	.002
Months with ACC contract, mean (SD)^b^				
CathPCI	88.5 (33.4)	98 (26.4)	83.92 (35.5)	<.001
ICD	101.1 (29.4)	106.8 (23.5)	98.36 (31.5)	<.001
Participation in PCI 30-d readmissions program	340 (22.2)	204 (42.1)	136 (13.0)	<.001
Composite discharge medications performance, mean (SD), proportion of hospitals				
CathPCI Registry	0.94 (0.06)	0.96 (0.03)	0.92 (0.07)	<.001
ICD Registry	0.84 (0.12)	0.88 (0.10)	0.81 (0.12)	<.001

Abbreviations: ACC, American College of Cardiology; CathPCI, catheterization and percutaneous coronary intervention; ICD, implantable cardioverter-defibrillator; PCI, percutaneous coronary intervention.

^a^ Among eligible hospitals participating or not participating by the end of 2016.

^b^ Until December 2015.

### Enrollment

There was an increase in hospital participation during the first year of the program, from 37 hospitals to 69 hospitals (86.5%). There was a greater increase in enrollment once data were available privately for hospitals to review and when they were made public. In March 2016, a month after the *USNWR* announcement of credit for participation in their hospital ranking system, the number of hospitals enrolled increased from 240 to 376 hospitals (56.7%) (Figure 1). By the end of 2016, of 1531 eligible institutions, 485 hospitals (31.8%) had elected to participate. Of these, 310 hospitals (63.9%) were enrolled in public reporting for CathPCI and ICD registries, while 175 hospitals (36.1%) participated in 1 registry. By May 2017, 561 of 1747 eligible hospitals (32.1%) had chosen to participate. Overall, more hospitals participated in the CathPCI registry (531 hospitals) than the ICD registry (374 hospitals). Among 561 participating hospitals, few (4 hospitals [0.7%]) dropped out of the program after the first year of public reporting.

### Discharge Medications and Hospital Performance

**Figure 2.**
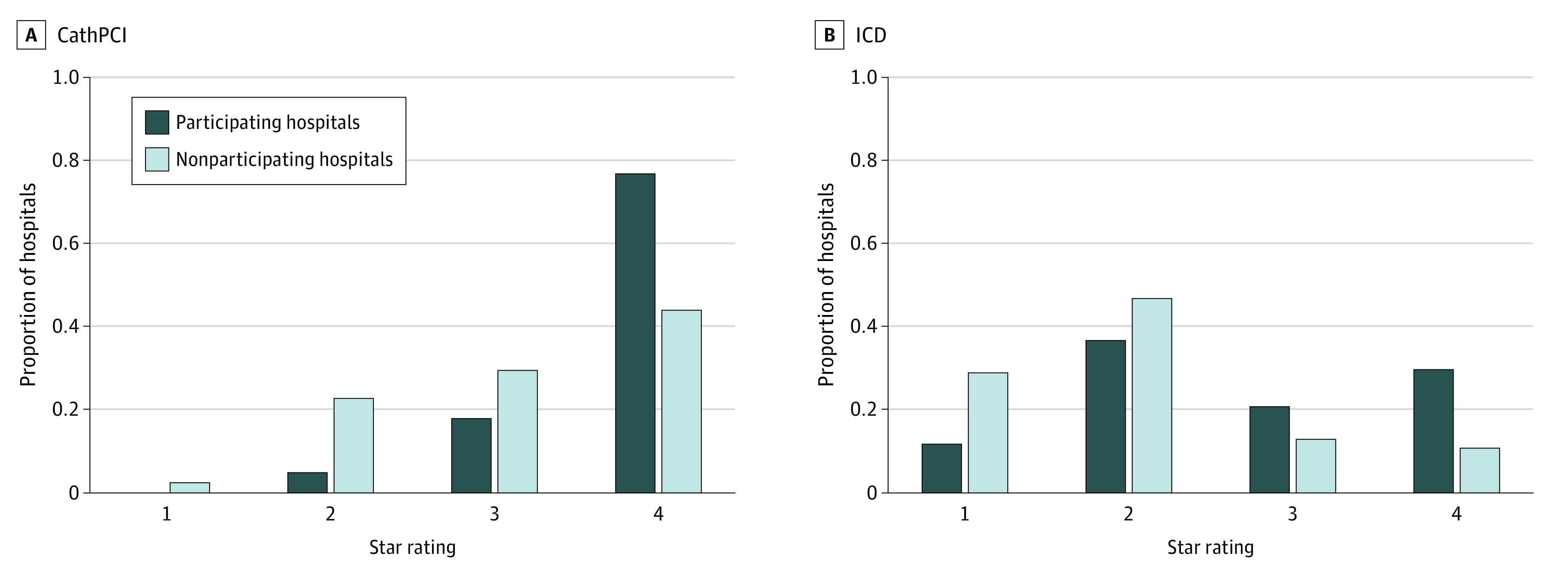
Participation in Voluntary Public Reporting by Hospital Star Ratings CathPCI indicates catheterization and percutaneous coronary intervention; ICD, and implantable cardioverter-defibrillator.

Hospitals participating in public reporting had increased mean (SD) proportion of hospitals with adherence to composite discharge medications after PCI (0.96 [0.03] vs 0.92 [0.07]; *P* < .001) and ICD (0.88 [0.10] vs 0.81 [0.12]; *P* < .001) (Table 2). Participating hospitals also had an increased proportion of 4-star (ie, highest) ratings compared with those that did not participate in CathPCI (372 of 483 hospitals [77.0%] vs 394 of 891 hospitals [44.2%]; *P* < .001) and ICD (121 of 408 hospitals [30.0%] vs 78 of 719 hospitals [10.8%]; *P* < .001) registries (Figure 2).

### Factors Associated With Participation in Public Reporting

**Figure 3.**
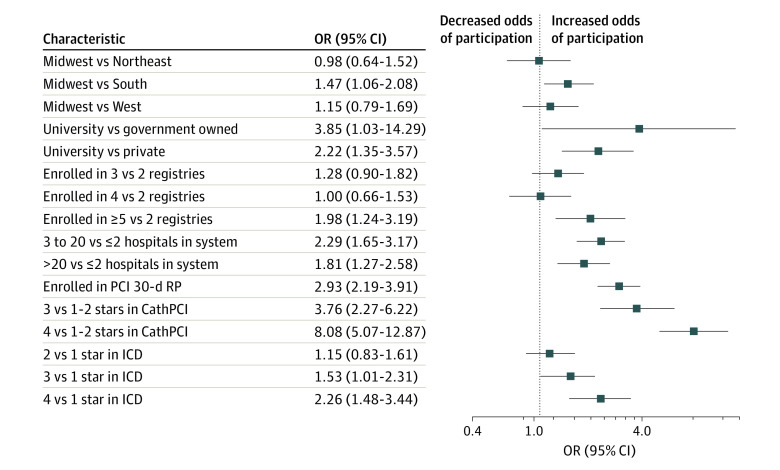
Characteristics Associated with Participation in Public Reporting CathPCI indicates catheterization and percutaneous coronary intervention; ICD, implantable cardioverter-defibrillator; OR, odds ratio; RP, readmissions program.

In multivariable analysis, several factors were associated with increased odds of participation in reporting. These included participation in 5 or more NCDR registries (odds ratio [OR], 1.98; 95% CI, 1.24-3.19; *P* = .005), membership in a larger hospital system (ie, 3 to 20 hospitals vs ≤2 hospitals in the system: OR, 2.29; 95% CI, 1.65-3.17; *P* = .001), enrollment in the PCI 30-day readmission public reporting pilot program (OR, 2.93; 95% CI, 2.19-3.91; *P* < .001), ownership by a university (vs government ownership: OR, 3.85; 95% CI, 1.03-14.29; *P* = .045; vs private ownership: OR, 2.22; 95% CI:1.35-3.57, *P* < .001), Midwest location compared with the South (OR, 1.47; 95% CI, 1.06-2.08; *P* = .02), and having an increased number of performance stars (4 vs 1-2 stars in CathPCI: OR, 8.08; 95% CI, 5.07-12.87; *P* < .001; 4 vs 1 star in ICD: OR, 2.26; 95% CI, 1.48-3.44; *P* < .001) (C statistic = 0.829) (Figure 3).

## Discussion

This cross-sectional study found that in the first 3 years after the launch of the ACC voluntary public reporting program for CathPCI and ICD registries, one-third of eligible hospitals opted to participate. Inclusion of the program in *USNWR* rankings was associated with an increase in participation. Compared with hospitals that chose not to participate, participating hospitals had increased procedural volume, performance ratings, experience with other public reporting programs, and adherence to composite discharge medications.

Despite increasing program enrollment in its earliest years, most eligible hospitals chose not to participate in the public reporting program. Some hospitals with low star ratings may have perceived no advantage to publicly reporting their performance owing to concerns that their reputation may have suffered.^15^ Other hospitals may not have been familiar with the reporting platform or may not have completely trusted the methodology used for public reporting and so feared consumer misinterpretation.^16,17^ In the case of the STS cardiac surgery voluntary reporting initiative, approximately 44% of eligible surgical programs opted in to public reporting in the first 5 years of the program.^15^ STS began providing confidential feedback to participant programs on their performance data 3 years before initiating voluntary public reporting. This period of confidential feedback and pilot testing may have been associated with increased trust among the programs in the validity of the process measures and increased motivation to participate.^15^

We found that the *USNWR* announcement that program participation would impact hospital’s national rankings was associated with an increase in enrollment. The increase in participation after the announcement suggests an important association of nonfinancial incentives linked to public perception and reputation with increased involvement in public reporting. Other incentives from regulators, payers, and government entities may also be essential in promoting participation in public reporting.^14^

Overall, characteristics common to larger hospitals and features that suggest a commitment to quality were more commonly seen in hospitals enrolled in voluntary public reporting. Participating hospitals were larger, affiliated to larger hospital systems, and more likely to be university owned and had an increased volume of PCI and ICD procedures. Larger hospitals with higher volume and teaching hospitals are historically more likely to publicly report outcomes.^15,18^ Several factors may be associated with these findings. First, large academic centers may have a greater interest in transparency and public reporting.^18^ Second, given the association of national hospital rankings with public perception, academic centers and larger hospitals may be more sensitive to opportunities that are associated with *USNWR* rankings.^19^ Participating hospitals were also more likely to be in states with existing public reporting programs and enrolled in an increased number of NCDR registries and the PCI 30-day readmission public reporting pilot program. These findings suggest that hospitals with an already-established structure, culture, and experience around quality improvement and public reporting may be more likely to participate in other similar initiatives.

Additionally, we found that higher-performing hospitals were more likely to participate in public reporting. Hospitals’ choices to report may be associated with knowledge of their performance results, as previously reported by an evaluation of programs enrolled in the STS public reporting program.^15^ In contrast, a prior study from the American College of Surgeons National Surgical Quality Improvement Program^18^ found that hospitals’ past and current performance was not associated with participation in their voluntary public reporting program. Our study findings suggest that potential ways to improve adoption of public reporting may include use of governmental and nongovernmental incentives that publicly reward participation, as well as promotion of public reporting as a culture of transparency that could influence public perception and reputation.

This first iteration of the ACC public reporting program represents one of the larger-scale national voluntary public reporting efforts, providing an assessment of compliance with process measures that are actionable and understandable. By using clinical data that accurately and fairly characterize the care delivered, this program strives to provide a more complete assessment of hospital performance while maintaining a single and interpretable rating. Furthermore, the NCDR public reporting program has already expanded to include 2 metrics from the Chest Pain-Myocardial Infarction (MI) Registry, and in the future, it may include risk-adjusted clinical outcomes and composite quality measures across NCDR registries.^9^

### Limitations

Our study has several limitations to consider. First, NCDR programs are voluntary, and so our results represent the practices only of centers reporting data to NCDR and may not be generalizable to all centers. However, because the study period predates the expiration of the Coverage with Evidence Development for ICDs that mandated submission of all Medicare cases to the ICD Registry, it captures most ICD implants.^20^ Likewise, more than 90% of PCI-capable hospitals in the United States participate in the CathPCI Registry.^21^ Second, while the number of eligible hospitals increased by 216 over the study period, from 1531 to 1747, this is not likely to explain the increased participation in the voluntary reporting program over time. Third, the detailed reasons why sites decided to participate or not in public reporting were not explored in this study. These should be further explored with qualitative research.

## Conclusions

One-third of eligible hospitals participated in the ACC voluntary public reporting program. Several hospital characteristics and experience surrounding quality measurement and public reporting were associated with participation, which had a substantial uptake after inclusion in national hospital rankings. Future studies may be warranted to increase adoption of public reporting and to assess characteristics of other public reporting initiatives from other registries, such as the Chest Pain-MI Registry.
